# The Study of the Impact of Empowering Leadership on Adaptive Performance of Faculties Based on Chain Mediating

**DOI:** 10.3389/fpsyg.2022.938951

**Published:** 2022-06-17

**Authors:** Ying Xu, Mengliu Zhang

**Affiliations:** School of Business and Management, Jilin University, Changchun, China

**Keywords:** LMX, leadership, psychology, performance, empowerment

## Abstract

High-quality faculties are the fundamental guarantee to achieving the connotation development of higher education. Hence, performing university faculties determines the quality of teaching and the level of talent cultivation. Facing the change in teaching demand and environment, faculties need to change their working methods spontaneously to achieve high-level performance. Relevant empirical studies have shown that empowering leadership positively affects adaptive performance. However, some researchers have found that leadership effectiveness even has a negative effect. There may be two reasons for the inconsistency in the effectiveness of empowering leadership: (1) There is a lack of in-depth research on the effectiveness of empowering leadership and employees’ performance in existing studies, and the exploration of its theoretical mechanism should be enriched. (2) The effectiveness of empowering leadership may be subject to the conditions of the individual’s characteristics of the empowering. Therefore, the mechanism of empowering leadership on faculties’ adaptive performance still needs to be further explored. This study explores the impact of empowering leadership on adaptive performance. Based on Social Exchange Theory and Psychology Empowerment theory, this study explores the mediating role of the leadership-member exchange relationship and psychological empowerment in the relationship between them. According to Regulatory Focus Theory, the moderating role of promotion focus and prevent focus was studied. We adopted questionnaire survey data including 292 individuals in Changchun, Shijiazhuang, and other cities; STATA 15 was conducted to test the hypotheses. The results showed that: (1) Empowering leadership was significantly and positively related to adaptive performance. (2) Leader-member exchange relationship and psychological empowerment play a mediating chain role in empowering leadership and adaptive performance; empowering leadership promotes psychology empowerment by enhancing the leadership-member exchange relationship, enhancing their adaptive performance. (3) Promotion focus positively regulates the relationship between psychological empowerment and adaptive performance. Individuals with a promotion focus have a significant positive impact on adaptive performance. Individuals with preventing focus do not weaken the positive impact of psychological empowerment on adaptive performance.

## Introduction

With the rapid development of China’s comprehensive national strength, China’s demand for talents increased. In recent years, talent management in colleges and universities has been paid more and more attention to experts and scholars and all walks of life. College teachers are the primary force to guarantee and improve the teaching quality in popular higher education. How to fully and effectively mobilize the creativity and enthusiasm of teachers and improve their performance level is also the core of human resource management in colleges and universities. Empowering leadership can give employees autonomy to a certain extent and encourage them to conduct behaviors conducive to work (Manz and Sims, 1987) spontaneously. Adaptive performance is the behavior at the individual level. It is the most effective behavior that can immediately respond to changes in the environment and change its behavior to meet the work requirements. It requires employees to adjust their work strategies (Jundt et al., 2015) flexibly. Although empirical studies have linked empowered leadership with adaptive behavior, how this leadership style promotes the adaptive performance of college teachers has not been fully revealed. The primary purpose of this study is to explore the relationship between allowed leadership and the adaptive performance of university teachers by establishing and testing a model.

According to social exchange theory (Lawler and Thye, 1999), the leader-member exchange relationship (LMX) can promote employees’ identification with the organization, internalize organizational goals into personal goals, maintain a high level of initiative and vitality at work (Tsui et al., 1997), and bring positive behaviors. Therefore, leadership behavior influences employees’ behaviors through the mediating role of their perception of social exchange relationships (Song et al., 2009). In addition, based on Thomas’ psychological authorization theory (Thomas and Velthouse, 1990), the influence of allowed leadership behavior on variables at the individual level of employees is influenced by employees’ psychological perception of authorization. Employees with high levels of psychological empowerment are more engaged and creative in their work, thus positively changing their behavior. Few studies have explored the mechanism of leader-member exchange and psychological empowerment on the relationship between allowed leadership and adaptive performance of university teachers, and the relationship between leader-member exchange and psychological empowerment is still questionable.

**FIGURE 1:**
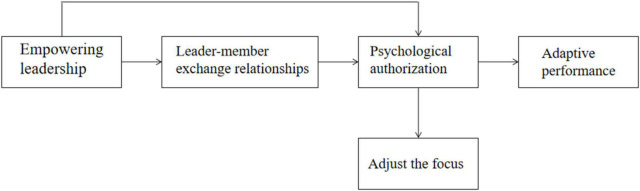
Theoretical model.

On the one hand, some scholars believe that leader-member exchange positively affects psychological empowerment (Song et al., 2009). Some scholars believe that the psychological empowerment level of employees at work can promote the positive emotional exchange between employees and leaders, thus forming a high-quality leader-member exchange relationship (Schriesheim et al., 1998; Mak and Sockel, 2001; Xu et al., 2018). Therefore, this research will be psychological authorization. The authorized leader-member exchange relationship between two variables is introduced into the leadership effect on performing university teachers’ adaptability in the model as shown in Figure 1, and added to adjust focus as a regulating variable. The author will also explore whether college teachers with unique characteristics have the same perception of psychological authorization and what kind of adaptive change they will make to their work behavior.

## Review and Hypothesis

### Adaptability Performance of College Teachers

Performance management refers to the continuous process of identifying, measuring, and developing individual and team performance and making these performances consistent with the strategic objectives of the organization (Gary, 1999). The two-factor Performance model of Task Performance and Contextual Performance proposed by Borman and Motowidlo (1993) is a classic model in Performance management. Task performance refers to the behavior directly related to the efficiency of the task stipulated by the organization. It is related to the core technical activities in the specific task; Relational performance refers to spontaneous behavior, organizational citizenship, organizational dedication, and performance behavior unrelated to specific tasks. It provides an organizational, social, and psychological environment for acquiring core technologies. Although the model can provide managers with direct performance evaluation methods, organizational change and environmental uncertainty make performance management the dynamic direction. Therefore, more and more scholars believe that the static two-factor performance model can no longer effectively cope with frequent and unexpected work requirements and environmental changes, and the adaptive behavior of employees should be considered in performance management (Allworth and Hesketh, 1999).

Allworth and Hesketh (1997) proposed for the first time in that it is necessary to pay more attention to the adaptive performance of employees to cope with changes based on the two-factor performance model to better pay attention to the response of employees to external changes (Wu and Denghua, 2010). Unlike the traditional static performance model, Adaptive Performance is generated by the constantly changing social environment and mainly refers to the behaviors of employees to cope with the changes. Individuals within the organization are required to have the ability to constantly learn new technologies and methods and be able to use creative thinking to effectively cope with environmental changes and meet job requirements (Allworth and Hesketh, 1997; Wu and Denghua, 2010). Pulakos et al. (2000) believe that adaptive performance comprises eight dimensions: creatively solving problems, dealing with uncertain work situations, coping with emergency or crisis events, learning new work techniques and methods, interpersonal adaptability, cultural adaptability, and coping with work pressure, and physical adaptability. For the college teachers, this study considers that adaptive performance is the behavior of teachers who actively respond to demands and environmental changes in college teaching and research work and achieve job requirements by actively designing and implementing new teaching and research programs.

### Empowering Leadership and Adaptive Performance

In an organization, many factors affect the ability and willingness of employees to make adaptive behaviors, and empowering leadership is one of them. Manz and Sim proposed that delegated leadership differs from the traditional top-down and up-handed leadership and emphasizes the initiative and consciousness of subordinates in their work. Therefore, empowering leadership is a behavior in which a leader distributes power to subordinates to improve their intrinsic motivation and change the cognition of their work roles (Manz and Sims, 1987; Srivastava et al., 2006).

Arnold et al. (2000) believe that empowering leaders can improve employees’ sense of self-efficacy by implementing a series of authorization processes. Creating external conditions can remove the influencing factors that lead to employees’ lack of autonomy, thus helping employees decide independently according to the work form. Since then, the research on the relationship between empowered leadership and employee job performance has become the focus of scholars (Arnold et al., 2000). Authorized leadership is committed to improving subordinates’ control over work and giving them enough space to give full play to their work planning and thinking. Therefore, authorized leadership behavior can also be regarded as redistributing resources and rights by leaders (Liu et al., 2003; Wang and Jianmin, 2018). Delegating leadership is good at delegating power and using the talents and wisdom of subordinates to achieve team goals. This behavior of delegating power can improve subordinates’ sense of responsibility at work and make them more responsible for their work behaviors and results. It consequently improves their efforts and problem-solving ability (Aryee and Chen, 2006), stimulating their desire to express opinions and suggestions (Srivastava et al., 2006) and keeping their thinking in an active state. On the one hand, subordinates will feel the leader’s recognition of their ability, thus improving their level of self-efficacy. On the other hand, in assuming responsibility and taking over the team, the creativity and desire of subordinates can be constantly stimulated, thus promoting employees to make more adaptive behaviors (Sergio and Iacobucci, 2010).

Many empirical studies have shown that empowering leadership has a significant positive impact on employee performance. However, some researchers have found that in certain situations, the effectiveness of delegated leadership may even have adverse effects (Zhang et al., 2018). Pang et al. (2013) discussed the inverted U-shaped non-linear relationship between authorized leadership and employee performance from four aspects: employee pressure, interpersonal conflict, peer pressure, and knowledge management. They believe that authorized leadership has a significant positive effect on employee performance, but excessive authorization is not beneficial but harmful, and employee performance will be reduced (Pang et al., 2013). The empirical study of Cheong et al. (2016) shows that empowering leadership can indirectly improve employees’ job role performance through self-efficacy, namely “enabling process,” and indirectly reduce employees’ job role performance through tension, namely “negative process”. Lin and Zhongheng (2017) analyzed the causes of over-authorization at the employee level and believed that the “too much of a good thing” effect exists in the authorized leadership. Excessive authorization causes increased work pressure, interpersonal conflict, and overconfidence, all of which harm employees’ work performance.

The theoretical inconsistency on the effectiveness of authorized leadership may be due to the following two reasons: (1) Existing studies lack in-depth research on the relationship between authorized leadership and employee job performance, and exploration of its mechanism should be enriched. (2) The effectiveness of empowering leadership may be limited by the personal characteristics of empowered individuals. In management practices, leadership must go through proper control and authorization to effectively manage the teachers in colleges and universities. It authorizes them to practice, convey confidence and autonomy, and promote participation should coexist, positively influencing college teachers’ adaptive performance, prompting teachers to adopt adaptive behavior to ensure that their efforts achieve results and achieve the desired goal. This study will elaborate on the mechanism of empowering leadership and employee job performance and the moderating role of personality traits in their relationship.

Therefore, the following hypothesis is proposed:

H1: Empowering leadership has a positive effect on adaptive performance.

### The Mediating Role of Leader-Member Exchange Relationship

Leader-member exchange relationships developed from social exchange theory play a crucial role in the process of employee behavior. In Dansereau et al. (1975), first proposed the leader-member exchange (LMX) theory. Leaders treat employees with different attitudes, and the intimacy between them is different, and their relationship is different. Employees are thus divided into “in” and “out” people. “In” employees get more trust and help from leaders than “outside” employees and have more opportunities to win more excellent resources.

The perspective of social exchange theory is based on the principle of reciprocity. When employees get rewards beyond the labor contract provisions at work, such as the care and support of leaders, the right to speak, autonomy in decision-making, higher organizational status, and self-esteem, employees will have the psychological reward. This kind of psychology will encourage employees to have a higher engagement in work and a greater motivation to learn new knowledge (Avolio et al., 2004). Therefore, the leader-member exchange relationship will change employees’ behavior, attitude, and psychology, and the influence of leadership behavior on employees’ positive behavior, attitude, and performance is generated through employees’ perception of the social exchange relationship as an intermediary variable (Song et al., 2009; Pieterse et al., 2010; Zhang et al., 2016). For college teachers, in a high-quality social exchange relationship, the authorization and support provided by leaders will make them more confident to complete challenging tasks. In the long run, college teachers will master superb working ability and more robust professional quality in the exchange process with leaders. Faced with students or unexpected situations, they are more determined and willing to achieve adaptive performance. Therefore, the following hypothesis is proposed:

H2: The leader-member exchange relationship mediates between authorized leadership and adaptive performance of university teachers.

### The Mediating Role of Psychological Authorization

The concept of psychological authorization develops along the motivation path of authorization and is formed from the research on self-efficacy authorization by Bandura (1977, 1982) and Conger and Kanungo (1988). It refers to employees’ cognitive state or perception, and subordinates can express themselves confidently at work. According to existing studies, Spreitzer (1995) proposed four parts of the psychological empowerment and intrinsic motivation concept: work meaning, self-efficacy, autonomy, and work influence. Work meaning refers to an employee’s work goals and objectives consistent with their beliefs or values. Self-efficacy is a belief in an employee’s ability to do a job within his or her capacity. Autonomy refers to the individual’s belief that he or she can choose how to start and adjust his or her behavior, which reflects the employee’s autonomy to start and continue his or her work behavior and procedures. Job impact refers to how an employee can influence an organization’s administrative, strategic, or operational outcomes. In addition, psychological authorization is also an incentive structure for employees to start and adjust their behaviors (Thomas and Velthouse, 1990; Spreitzer, 1995).

Spreitzer believes that the influence of individual/organizational factors on individual/organizational results is realized through the mediating role of psychological authorization (Spreitzer, 1995), and leadership style has a limited influence on psychological authorization through standards, norms, rules, and policies (Pieterse et al., 2010). The authorization behavior of an authorized leader does not directly affect employee behavior. Only when employees perceive the improvement of the leader’s authorization level, and such leadership behavior affects their work significance, autonomy, work influence, and self-efficacy, can employee behavior change. The higher the level of psychological authorization, the stronger the psychological return of employees to the organization, and the better the promotion effect on employees’ behavior (Tsui et al., 1997; Song et al., 2009; Zhang and Bartol, 2010). Through investigation and analysis of 377 MBA students, JiaTao HUANG found that psychological authorization leads to positive behaviors in employees, and employees with solid cognition of authorization are more likely to show positive behaviors than other employees (Jiatao, 2017). Jundt made a detailed review of the adaptive performance of individuals in organizations and divided the influencing factors of adaptive performance into two categories: (1) Personal factors and situational factors, (2) motivational factors, and knowledge-based factors (Jundt et al., 2015). The former are long-term factors, reflecting the characteristics of the individual/work/task environment, which are relatively stable over time and from individual to individual. In contrast, the latter are near-term factors that directly affect performance. As a motivational factor, self-efficacy positively affects adaptive performance.

Therefore, the following hypothesis is proposed:

H3: Psychological empowerment mediates between empowering leadership and adaptive performance.

Social cognition theory proposes that people’s understanding and interpretation of their practices and ideas will also be different in different environments. In a team, leading member exchange can be a feature of the social structure. Psychological empowerment reflects individual role orientation, and some studies have proved that high-quality leader-member exchange relationships can promote psychological empowerment (Aryee and Chen, 2006; Dulebohn et al., 2011; Zhou et al., 2012). When employees gain the right to solve problems, learn more information and get more support at work through high-quality exchange relationships, they will perceive a higher level of psychological empowerment (Koberg et al., 1999). Kwak and Jackson (2015) proposed that empowered leaders influence employees’ psychological empowerment through the high-quality working relationship that employees think is formed between themselves and their leaders. A leader who wants to maximize employee empowerment may consciously establish different working relationships with employees in a group. When leaders engage in empowering behavior in their relationships with employees, employees may increasingly perceive high-quality leader-member exchange relationships. High-quality leader-member relationships perceived by employees can further positively affect the four job perceptions that reflect employees’ empowerment at work. The leader-member exchange can play an essential role as a potentially crucial intermediary mechanism to convey the indirect influence of authorized leadership on employee psychological empowerment, surpassing the direct influence of authorized leadership. Therefore, the following hypothesis is proposed:

H4: Leader-member exchange has a positive impact on psychological empowerment.

H5: Authorized leadership influences the adaptive performance of college teachers through the positive effect of the leader-member exchange relationship on psychological authorization.

### Adjust the Focus

When the external environment changes, people will adjust their attitudes and behaviors to make coping strategies, and such adjustment is based on the role of adjusting focus (Fredrickson and Losada, 2005). Higgins first proposed the regulatory focus theory in 1997 (Crowe and Higgins, 1997). He believes individuals will constantly self-regulate to combine their status (including behaviors and thoughts) with changing goals or standards when facing different goals or standards. The characteristics of seeking benefits and avoiding harm are mainly derived from different regulatory focuses, which makes people have differences in thinking and doing things. According to the different goals, the regulatory focus can be divided into the following two types: The first is the promotive regulatory focus, which makes people more sensitive to the reward acquisition behavior; they pay more attention to the positive goals; The second is the defensive regulatory focus, which makes people more sensitive to punishment avoidance behavior, they pay more attention to the hostile target. Accelerative regulatory focus focuses people on positive outcomes and motivates them to work hard to achieve the desired outcome. The defensive change focus makes people pay more attention to the possibility of failure, avoid deviating from the “track” alertly, and follow the rules to prevent failure (Shi, 2010; Li and Yufan, 2011).

Michele and Tucker (2016) proposed that defensive regulatory focus can promote good tactics in impression management, promoting regulatory focus can promote pleading tactics, and leadership behavior integration plays a positive moderating role. In psychological empowerment affecting work performance, individuals with facilitative regulatory focus identify with the organization and themselves from the heart after experiencing happiness. They are more likely to adopt a positive attitude and constantly adjust their work methods to achieve high performance. Defensive adjustment focus on individuals who are less sensitive to negative results. Based on safety needs, they often take negative work behaviors to avoid negative results caused by failure. When previous work skills are no longer suitable for the needs of the job, the facilitator will actively seek new work ideas and attempt to achieve the ultimate success. Defensive adjustment focuses on individuals who are resistant to learning and applying new work skills because it means unpredictable results and the possibility of failure.

In the past, it was believed that there was a significant difference between the stimulative and defensive regulatory focus on individual innovation behavior. However, the current research on the role of the defensive regulatory focus is controversial. When exploring how expectation evaluation affects employee creativity, Wang et al. (2017) found that stimulative regulatory focus individuals are more creative when evaluating expectation information. In contrast, defensive regulatory focus individuals are more creative when evaluating expectation control. The role of both accelerative and defensive regulatory focus depends on whether it is maladjusted. The goal is achieved and successfully or not achieved and actively continued to be achieved. For the individual with a defensive regulatory focus, when the individual has defensive emotions (fear, Etc.) or the goal is not accomplished, the defensive regulatory focus is activated, and the individual generates many ideas, insights, and solutions to problems. The defensive regulatory focus is not activated when the individual achieves the defensive goal, and the regulatory focus disorder (Baas et al., 2011).

As a particular group, college teachers are distinguished from other social workers by their academics, independence, and autonomy (Li et al., 2012). College teachers have internal solid motivation needs and need psychological satisfaction in their work to stimulate internal motivation. Some scholars believe that the intrinsic work motivation of college teachers comes from the motivation factors that stimulate behavioral motivation. It includes teachers’ sense of achievement, responsibility, ability and interpersonal relationships, Etc., which is a more lasting and powerful motivation with inherent spontaneity and autonomy (Gao, 2001; Cheng, 2004). Internal motivation favors self-efficacy and self-control. Its applicability is higher in the face of college teachers’ innovative and challenging work, which is conducive to achieving job performance (Teng et al., 2018).

College teachers have the proactive personality traits of flexibility and adaptability in uncertain situations, with strong intrinsic motivation and higher autonomy and initiative. Different teachers may have different responses to the same environmental changes due to the difference in the level of active personality, but the goal is to pursue high performance. The change in work demand and environment triggers the intrinsic motivation of college teachers. Teachers with a high level of dynamic personality have activated the focus of stimulative regulation, dare to take responsibility, and will try all kinds of methods to solve problems actively and achieve goals. For teachers with a relatively low level of proactive personality, when the goal is not completed, the defensive adjustment focus is activated to choose how to avoid the risk of failure and actively choose other ways to achieve a high level of performance.

Therefore, the following hypothesis is proposed:

H6: Accelerative regulatory focus can strengthen the influence of psychological empowerment on adaptive performance.

H7: Defensive regulatory focus can strengthen the influence of psychological empowerment on adaptive performance.

## Research Methods

### Research Samples and Procedures

This study mainly distributed questionnaires to university teachers in Changchun, Shijiazhuang, and other cities. The Data was collected by sending written questionnaires to their sites, e.g., Universities, labs, Offices, Etc. After the questionnaires were filled in, researchers recycled and sealed them. In this survey, 400 questionnaires were sent out, 373 were recovered, 81 invalid questionnaires were excluded, and 292 valid questionnaires were kept, with a functional recovery of 78.28%. The demographic results for the samples are: The male to female ratio is 102–190, close to the level of 1:2. Most of them were 25–41 years old, with an average age of 33.11 (*SD* = 4.952). Regarding education level, 251 students had a bachelor’s degree or above, accounting for 86.30% of the total number of subjects, which showed that the data source of this study was highly credible, and the subjects could solve the questions and select their answers effectively.

### Measurement of Variables

In this study, the main variables were authorizing leadership, leader-member exchange, psychological empowerment, adaptive performance, and regulatory focus. To ensure the content validity of the study, all entries originally in English were translated into Chinese by two bilingual (English-Chinese) scholars. All scales were based on the Likert five-point scale, ranging from 1 “strongly disagree” to 5 “strongly agree.”

1. Authorizing leadership. Ahearne et al.’s (2005) scale was used to measure authorized leadership, divided into four dimensions: clear job meaning, encouragement to take part in decision-making (promotion), confidence in subordinates’ ability, and provision of job autonomy. Each dimension had three questions, a total of 12 questions. They are, respectively, as follows; “My boss helps me understand the relevance of my goals to the company’s goals.” “My boss helps me realize how important my work is to the big picture.” “My boss helps me understand how my work fits the big picture.” “My boss often involves me in decision-making.” “My boss often discusses strategic decisions with me.” My boss will ask me about the idea in advance. “my boss believes I can deal with complicated work.” “even make a mistake, my boss still believes I can progress and improve.” “my boss fully believes I can complete the task well.” “my boss allows me to do things in its way.” “my boss will keep the rules and regulations as concise as possible,” and “My boss allows me to decide quickly to meet customer needs.” The Cronbach’s α coefficient of the scale in this study was 0.93.
2. Leader-member exchange. The most classic leadership member exchange measurement scale is the one-dimensional measurement scale developed by Graen and Uhl-Bien (1995). Since this study uses the classic LMX-7 scale to measure the leader-member exchange relationship, there are seven items in total. “I have always known what to do to meet my boss’s needs.” “My boss understands the difficulties and needs of my job.” “My boss can reach my potential.” “My boss uses personal resources to help me solve problems at work.” “My boss forgives my mistakes.” “I will defend and justify my boss’s decisions.” “I think I have a very close working relationship with my boss.” The Cronbach’s α coefficient of the scale in this study is 0.88.
3. Psychological empowerment. This study uses the psychological empowerment scale developed by Spreitzer (1995), divided into four dimensions, including meaning, competence, self-determination, and influence. There are three questions for each dimension and a total of 12 questions, which are, respectively; “The work I do is significant to me”; I have great confidence in my ability to get the job done; “I have a great deal of autonomy in deciding how to get the job done,” and “I have a great deal of influence over what happens in my department.” “The Cronbach’s α coefficient of the scale in this study was 0.93.”
4. Regulatory focus. An 18-item scale (GRFM Scale) developed by Lockwood et al. (2002) was used to measure regulatory focus. The GRFM scale developed by Higgins et al. (2001) differs from the traditional RFQ scale. It directly measures the decision-making tendency of the subjects toward pursuing success and the avoidance of loss, and the measurement result is the tendency of the subjects toward positive and negative results. Therefore, GRFM reflects the current result state. The scale contains two factors, one is the stimulative regulatory focus, and the other is the defensive regulatory focus. There are nine questions for each factor. The items of accelerative regulatory focus are: “I often think about how to realize my ambition,” “I often dream about my ideal self,” and “I often think about how to make myself successful,” Etc. Cronbach’s α coefficient of this scale in this study is 0.91. The items of the defensive adjustment focus target are: “I carefully avoid negative influences in life,” “I am apprehensive about dereliction of duty at work,” and “I often think about what I am afraid of happening,” Etc. Cronbach’s α coefficient of this scale in this study is 0.90.
5. Adaptive performance. After a systematic study, Pulakos et al. (2000) proposed that adaptive performance should contain eight dimensions. Domestic scholars (Zhang and Quanquan, 2009), based on Pulakos’ research, got the adaptive performance scale suitable for teachers, including seven dimensions of cultural promotion, active problem solving, stress handling, emergency handling, interpersonal promotion, continuous learning, and physical adaptation, with 40 items. This study uses this scale to measure the adaptive performance of college teachers. It includes “keeping calm when work pressure is too high,” “thinking clearly and prioritizing when dealing with urgent problems,” “Working well with people with different personalities,” “Learning new knowledge or skills quickly,” and “solving complex problems innovatively,” and “quickly applying new procedures or tasks.” The Cronbach’s α coefficient of the scale in this study was 0.85.

## Research Results

STATA 15 was used for data analysis in this study. First, confirmatory factor analysis was used to test the discriminative validity of this study. Second, descriptive statistical analysis was carried out on the samples through correlation analysis, and the correlation between variables was tested. Finally, the mediating effect and moderating effect are tested by hierarchical regression and structural equation model.

### Confirmatory Factor Analysis

In this study, STATA 15 software was used to conduct confirmatory factor analysis to test the discriminative validity of the model (six latent variables were authorized leadership, psychological authorization, leader-member exchange, adaptive performance, facilitative regulatory focus, and defensive regulatory focus). At the same time, the model fitting index is compared with other alternative models, and the results are shown in Table 1. The discriminative validity of the six-factor model was good, χ^2^ = 1613.40, CFI = 0.969, TLI = 0.967, SRMR = 0.043, RMSEA = 0.025, and the model fit was good. Compared with the other five alternative models, the fitting effect is better. The model has better structural validity and can be tested in the next step by the structural equation model.

**TABLE 1:** Results of confirmatory factor analysis.

Measurement model	χ^2^	df	Δχ^2^	CFI	TLI	SRMR	RMSEA
1. Six-factor model	1613.40	1,362		0.969	0.967	0.043	0.025
2. Five-factor model	2484.36	1,367	870.96**	0.860	0.854	0.093	0.053
3. Four-factor model	4017.44	1,371	2404.04**	0.669	0.655	0.118	0.081
4. Three-factor model	4492.03	1,374	2878.63**	0.610	0.594	0.124	0.088
5. Two-factor model	5467.81	1,376	3854.41**	0.488	0.468	0.136	0.101
6. Single-factor model	6029.63	1,377	4416.23**	0.418	0.395	0.138	0.108

n = 292. LMX represents leader-member exchange. Model 2 included authorized leadership + LMX, psychological authorization, adaptive performance, facilitative regulatory focus, and defensive regulatory focus. Model 3 included authorized leadership + LMX + psychological authorization, adaptive performance, facilitative regulatory focus, and defensive regulatory focus. Model 4 included authorized leadership + LMX + psychological authorization + adaptive performance, facilitative regulatory focus, and defensive regulatory focus. Model 5 was emollient leadership + LMX + psychological authorization + adaptive performance, facilitative regulatory focus + defensive regulatory focus. Model 6 was a single-factor model, combining all variables. Δχ^2^ was correlated with model 1. **p < 0.01.

### Descriptive Statistical Analysis

The descriptive statistical analysis results and correlation coefficient matrix of the main variables in this study are shown in Table 2. As shown in Table 2, empowering leadership and adaptive performance (*r* = 0.14, *p* < 0.05), leader-member exchange (*r* = 0.30, *p* < 0.001), psychological authorization (*r* = 0.25, *p* < 0.001), which? all showed a significant positive correlation. The relationship between leader-member exchange and psychological empowerment (*r* = 0.30, *p* < 0.001), adaptive performance (*r* = 0.20, *p* < 0.001). They are all positive correlations. Psychological empowerment and adaptive performance (*r* = 0.27, *p* < 0.001)are positive correlation. The results of correlation analysis preliminarily verified the relationship between the variables mentioned in the hypothesis and provided a basis for the data analysis in the next step.

**TABLE 2:** Descriptive statistical results and correlation coefficient matrix.

Variable	Mean	Standard deviation	1	2	3	4	5	6	7	8	
Gender	0.35	0.48	–								
Age	33.11	4.95	0.07	–							
Record of formal schooling	0.68	0.47	0.08	–0.02	–						
Empowering leadership	4.66	0.53	0.04	–0.07	0.00	(0.93)					
LMX	4.68	0.55	0.00	–0.04	0.03	0.30***	(0.88)				
Psychological authorization	4.66	0.53	0.02	–0.09	–0.02	0.25***	0.30***	(0.93)			
Accelerative regulatory focus	4.66	0.53	0.02	0.02	0.06	0.35***	0.60***	0.22***	(0.91)		
Defensive adjustment focus	4.68	0.52	0.07	–0.02	0.06	0.23***	0.35***	0.50***	0.32***	(0.90)	
Adaptive performance	4.67	0.57	0.09	0.04	0.04	0.14*	0.20***	0.27***	0.17**	0.18**	(0.85)

*n = 292. LMX represents a leader-member exchange relationship. The cronbach (α) coefficients are in parentheses.*

*p < 0.05; **p < 0.01; ***p < 0.001, Bilateral inspection.

### Hypothesis Testing

Multiple regression analysis was conducted through STATA 15, and the verification results are shown in Table 3. First, a structural equation model was constructed with authorized leadership as the independent variable and adaptive performance as a dependent variable. It can be seen from M4 that, after controlling for gender, age, and educational background, authorized leadership has a significant positive effect on adaptive performance (β = 0.13, *p* < 0.05). it verifies the hypothesis of H1. Second, when the leader-member exchange was introduced in M5, the positive effect of authorized leadership on adaptive performance was reduced (β = 0.08, *p* > 0.05), and the leader-member exchange had a significant positive effect on adaptive performance(β = 0.18, *p* < 0.01), hypothesis H2 is verified. When psychological empowerment was introduced into M6, the positive effect of empowering leadership on adaptive performance was reduced(β = 0.07, *p* > 0.05). at the same time, psychological empowerment had a significant positive effect on adaptive performance (β = 0.25, *p* < 0.001), and hypothesis H3 was tested. M2 showed that leader-member exchange had a significant positive effect on psychological empowerment (β = 0.30, *p* < 0.001), verifying the hypothesis of H4.

**TABLE 3:** Regression analysis results.

Variable	Psychological authorization		Adaptive performance						
		
	Model 1	Model 2	Model 3	Model 4	Model 5	Model 6	Model 7	Model 8	Model 9
**Control variables**									
Gender	0.03	0.03	0.09	0.09	0.09	0.08	0.08	0.09	0.08
Age	–0.09	–0.08	–0.04	–0.03	–0.03	–0.01	–0.02	–0.02	–0.02
Record of formal schooling	–0.03	–0.04	0.03	0.03	0.03	0.04	0.03	0.03	03
**The independent variables**									
Empowering leadership				0.13*	0.08	0.07	0.04	0.03	–0.05
LMX		0.30***			0.18**		0.12*	0.09	0.04
Psychological authorization						0.25***	0.23***	0.22**	0.29***
Accelerative regulatory focus Defensive regulatory focus								0.01 0.05	0.02 0.03
**Interaction**									
PE X Accelerative regulatory focus PE × Defensive regulatory focus									0.13* 0.18**
*R* ^2^	0.01	0.10	0.01	0.03	0.06	0.09	0.10	0.10	0.15
Adjust *R*^2^	–0.00	0.09	0.00	0.02	0.04	0.07	0.08	0.08	0.12
Δ*R*^2^		0.09***		0.02*	0.03**	0.06***	0.07***	0.00	0.04**

n = 292. LMX represents a leader-member exchange relationship. M5\M6\M7 of ΔR^2^ are all relative to M4, *p < 0.05, **p < 0.01, ***p < 0.001, Bilateral inspection.

**FIGURE 2:**
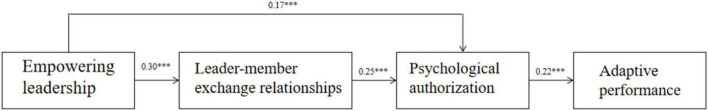
Mediating action path diagram of structural. *** means *p* < 0.001.

In order to reveal the mediating chain effect of the leader-member exchange relationship. Moreover, psychological authorization on the relationship between authorized leadership and adaptive performance, this study got the chain mediating path diagram by constructing a structural equation model H5 passed the test (see Figure 2).

**FIGURE 3:**
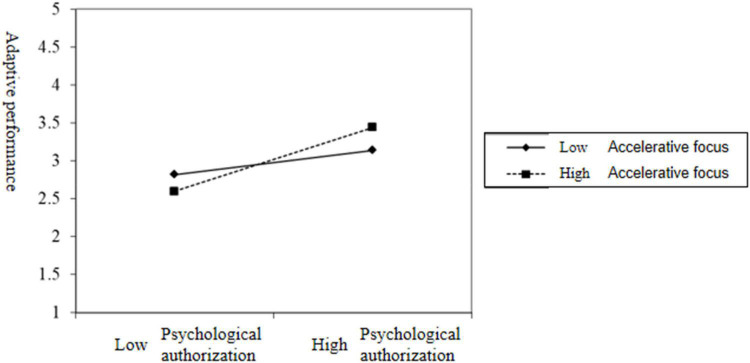
The moderating effect of facilitative moderating focus on psychological empowerment and adaptive performance.

**FIGURE 4:**
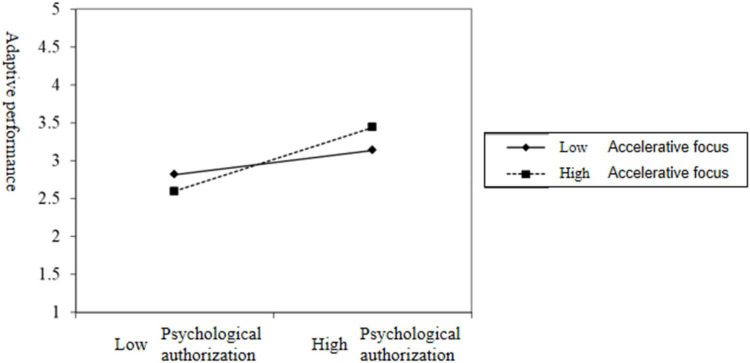
The moderating effect of defensive regulatory focus on psychological empowerment and adaptive performance.

Finally, to test the moderating effect of moderating focus, this study constructed the interaction term between psychological authorization and moderating focus, and the test result is M9 in Table 3. The interaction term between psychological authorization and facilitative regulatory focus had a significant positive effect on adaptive performance(β = 0.13, *p* < 0.05), and the interaction between psychological authorization and defensive adjustment focus also had a significant positive effect on adaptive performance(β = 0.18, *p* < 0.01). Therefore, the hypothesis of H6 and H7 have been tested. In order to more intuitively show the moderating effects of accelerative and defensive regulatory focus on the relationship between psychological empowerment and adaptive performance are shown in Figures 3, 4, respectively.

## Discussion and Theoretical Contributions

Empowering leadership has been proven to have a significant impact on adaptive performance. This study explores the mediating chain effect of the relationship between empowering leadership and adaptive performance and the moderating effect of moderating focus. The results show that authorized leadership positively affects college teachers’ adaptive performance, supporting the existing research. The leader-member exchange relationship and psychological authorization play a mediating role between them. Based on reciprocity theory and psychological empowerment theory, this study finds that subordinates will work harder to establish high-quality leader-member exchange relationships to live up to the trust of leaders. Once a high-quality leader-member exchange relationship is achieved, the extra efforts made by college teachers to maintain a high-quality relationship also make them believe that they have a significant impact on their work. Therefore, psychological empowerment will affect the adaptive performance of college teachers and make them adjust their work strategies.

Unlike previous studies, the empirical results also show that both facilitative and defensive moderating focus can enhance the positive effect of psychological empowerment on adaptive performance. The moderating effects of the two are not significant. The reason is that psychological authorization is an individual’s psychological perception of authorization and an individual’s internal motivation. The strength of this internal motivation will affect the enthusiasm and initiative of employees in the enterprise and then affect their work performance. Compared to other social groups, the university teachers’ group has a higher intrinsic motivation. When the authorization of the psychological experience higher teachers think of their work has to influence, confidence in the ability to work, the lower level of perception of external restrictions in the uncertain environment showed better adaptability, more actively to complete performance targets. In addition, as a research hotspot in motivation, regulatory focus theory focuses on the individual’s self-regulation of the desired last state. It is one of the personality theories with an important influence on exploring individual behavioral motivation, emotional experience, and behavioral performance. According to the regulatory focus theory, the accelerative regulatory focus is more concerned with progress, growth, and achievement.

In contrast, the defensive regulatory focus is more concerned with security and responsibility. Therefore, individuals with accelerative regulatory focus tend to desire success, adopt approach matching strategy, and do not miss any opportunity. Individuals with a defensive regulatory focus tend to focus their attention and adopt strategies to avoid mismatches to ensure that no mistakes are made to achieve their goals (Crowe and Higgins, 1997). Liu et al. (2017) found through empirical research that when individual motivation is activated, both the stimulative and defensive regulatory focus will lead to higher performance. The professional characteristics of college teachers are unique, and they bear the great responsibility of “teaching and educating people.” Based on the basic idea of being a model for teachers, teachers’ words and deeds will affect students’ outlook on life and values. When college teachers perceive psychological authorization as high, their defensive adjustment focus is more activated because of their powerful sense of responsibility. Therefore, they tend to choose strategies to avoid mismatching their goals and actively engage in their work to make progress while maintaining stability to get higher performance and provide a stronger guarantee for students and their development.

This study makes some unique contributions. First, the results are consistent with most other studies, and there is a positive correlation between empowering leadership and adaptive performance. The overall contribution is to establish and verify college teachers’ authorized leadership and adaptive performance model and introduce the leader-member exchange relationship and psychological authorization into the model. In the past, leader-member exchange and psychological empowerment were used as mediating variables to explore the mediating mechanism. In this study, both leader-member exchange and psychological authorization were used as mediators to verify the process of authorization-psychological perception-behavior and reveal the chain mediation process of authorizing leadership affecting adaptive performance. This can make the psychological process more visualized, three-dimensional, and involving variables indispensable. Therefore, this study explores the mediating mechanism of the relationship between authorized leadership and college teachers’ adaptive performance from a psychological perspective, especially the relationship between leader-member exchange and psychological authorization, which is a significant expansion of this research field and a supplement to existing research.

Secondly, different from previous studies, this study creatively combines social exchange theory and psychological empowerment theory to explore the relationship between leader-member exchange and psychological empowerment. It is found that high-quality leader-member exchange can enable employees to obtain more resources and information. Employees can obtain more work support through the positive behavior of leaders and thus perceive a higher level of psychological empowerment. Therefore, this study further verifies the positive impact of leader-member exchange on psychological empowerment, showing that positive reciprocity is critical in deepening employees’ perception of psychological empowerment. This study deepens the research on the effectiveness of leader-member exchange and expands the research scope of psychological empowerment theory.

Finally, another important theoretical implication is that this study, based on the moderating focus theory, verifies that moderating focus can moderate the effect of psychological empowerment on adaptive performance. Previous studies have confirmed the positive effect of psychological empowerment on adaptive performance, but they have ignored that unique personality traits regulatory focus influence this positive effect. Cognitive, motivation, impulsivity, emotion, and feedback have focused on regulatory focus research. In this study, the facilitative moderating focus plays a positive role in strengthening the psychological empowerment of college teachers on adaptive performance. In contrast, the defensive moderating focus does not weaken this result. Thus, from the perspective of model construction as a whole, the moderating effect makes the model more illustrative. With the increase of differences in psychological characteristics, this research model is more comprehensive and convincing in describing the formation of the entire process. This innovation promotes the research progress of moderating focus theory and has important implications for the further integration of moderating focus theory with other types of social exchange relations in the future.

## Management Enlightenment

In this study, we introduce the leader-member exchange relationship and psychological authorization as the continuous mediating variables of the relationship between authorized leadership and college teachers’ adaptive performance and introduce the moderating variable of moderating focus to adjust the relationship between psychological authorization and adaptive performance. This theoretical model is also very enlightening to university administrators.

First of all, authorized leadership can promote adaptive performance through the power of exchange between leading members and the psychological authorization of university teachers. Colleges and universities can offer training courses on how this process works. In addition, training courses should also include lessons on how to improve the level of leadership exchange within the teacher team and teach leaders the methods and skills to turn outsiders into insiders quickly. Leaders are encouraged to understand the individual needs of different teachers and increase their emotional input so that leaders can understand the mechanism behind social exchange at the theoretical level and grow and make breakthroughs in practice. At the same time, leaders should be especially reminded in the training process that the cultivation and encouragement of “insiders” need to be careful, adhere to the principle of fairness and justice, create a fair management atmosphere, and create an excellent working atmosphere of active efforts and struggle.

Second, the management needs to moderately change the management mode and strive to create an authorized management style. The teachers in colleges and universities are getting younger and younger, while the new generation of teachers has significant changes in their working and communication modes. They pursue self-development, individuality, independence, and strong innovation ability. Therefore, management methods should be changed accordingly. Research results show that delegation management is a very effective leadership behavior. In their work, leaders should often emphasize to teachers, especially young teachers, the value of their work, the help for future career development, and the realization and sublimation of self-value. It will make teachers feel more consistent with their work and values, thus enhancing their loyalty to work and motivation for work. In addition, leaders should delegate power appropriately. Young teachers have a strong sense of innovation and have many fresh ideas. While encouraging them to take part in decision-making, they will also create new possibilities for the development of colleges and universities.

Third, leaders should pay attention to improving the psychological authorization level of the team while constantly improving their abilities. Leaders need to improve their abilities because out of the worship and respect for leadership; subordinates can be more motivated in the team to bring higher work performance. In addition, leaders also need to explore other methods, such as organizing spring outings and teachers’ sports meetings, to make college teachers feel more psychological authorization to improve their perception of work significance. Leaders should also pay attention to the reasonable allocation of moderate difficulty tasks for different teachers so that teachers can enjoy the joy of success when facing challenges and breaking through themselves and establish stronger self-confidence for themselves.

Finally, leaders should observe the characteristics of teachers with unique personality traits and maximize teachers’ intrinsic motivation. For teachers with the characteristics of accelerative regulatory focus, leaders should actively create an atmosphere of innovative problem-solving. Leaders need to create a relaxed environment that encourages teachers to be innovative and adventurous and create an atmosphere of active thinking and free exploration. Driven by such an environment, the individual with a promotive regulatory focus can maximize the transformation of intrinsic motivation into actual proactive behavior. For teachers who defensive regulatory focus, when work demands and the environment change, leaders should help them analyze the size of risks and how to avoid risks and achieve higher performance effectively.

### Deficiencies and Prospects of Research

(1) This study only considers the positive effect of empowering leadership. Some scholars believe that besides bringing work resources and improving employees’ work motivation, authorized leadership may also have adverse effects, such as certain work pressure and physical and mental health problems (Borman and Motowidlo, 1993). In addition, excessive authorization will also lead to individuals’ psychological and behavioral avoidance behaviors (Jundt et al., 2015; Jiatao, 2017). Therefore, future research can be carried out on the boundaries of authorizing leadership.
(2) Leader-member exchange is a variable that changes over time, but this study is not a long-term analysis. It investigates data from a single time node and lacks dynamic data sources. If future studies can use dynamic data to measure relationships with other variables, the results will be more aim. In addition, this study can use the experimental method to observe the subjects’ behavior for a long time, which makes the research method more sufficient and the results more convincing.
(3) There is still much room for selecting dependent variables. For example, we can continue to study other variables, such as job satisfaction at the individual level, engagement, and organizational citizenship behavior. Scholars can also choose organization-level variables as the dependent variables of their research, such as organizational performance, innovation, and organizational learning climate. In addition, this study explores the influence of psychological empowerment as a mediating factor in leader-member exchange relationships and adaptive performance. Scholars need to continue exploring other mediating variables that play a role in this process to enrich the research results.
(4) The results of sample extraction have limitations. Although most data sources in this study are concentrated in northern China, individual differences caused by the region are not considered. Therefore, sample selection should be more random and universal in future studies.

## Data Availability Statement

The original contributions presented in this study are included in the article/supplementary material, further inquiries can be directed to the corresponding author.

## Author Contributions

Both authors listed have made a substantial, direct, and intellectual contribution to the work, and approved it for publication.

## Conflict of Interest

The authors declare that the research was conducted in the absence of any commercial or financial relationships that could be construed as a potential conflict of interest.

## Publisher’s Note

All claims expressed in this article are solely those of the authors and do not necessarily represent those of their affiliated organizations, or those of the publisher, the editors and the reviewers. Any product that may be evaluated in this article, or claim that may be made by its manufacturer, is not guaranteed or endorsed by the publisher.
